# Discursive delegitimisation of homosexuality on Chinese social media

**DOI:** 10.3389/fpsyg.2023.1178572

**Published:** 2023-09-12

**Authors:** Ke Zhang, Huibin Zhuang

**Affiliations:** School of Culture and Communication, Shandong University, Weihai, China

**Keywords:** China, homosexuality, Sina Weibo, discourse analysis, delegitimation strategies, sociocultural factors

## Abstract

This article contributes to the understanding of public representations of homosexuality in China by focusing on the case of a homophobic textbook. College student Xixi sued Jinan University Press (JUP) in 2017 for classifying homosexuality as a psychosexual disorder. Three years later, a Chinese court dismissed Xixi’s lawsuit against the allegedly homophobic textbook published by JUP. The ruling elicited responses on Chinese social media that demonstrated the polarisation of public opinion regarding homosexuality. This article investigates discursive representations of homosexuality in online space by analysing the public discourse surrounding this problem. Using van Leeuwen’s discursive delegitimation strategies (i.e., authorisation, moral evaluation, rationalisation, and mythopoesis), 496 comments posted on Sina Weibo were employed and subjected to discourse analysis. According to our findings, these strategies contribute to public opposition to homosexuality, portrayed as unhealthy, infertile, disruptive, and corrosive. The article concludes by discussing the emerging sociocultural factors on Sina Weibo that influence the anti-homosexuality attitudes of Weibo users.

## Introduction

On 17 May 1990, the World Health Organisation removed homosexuality from the list of mental illnesses. Following this, in 2001, the third edition of the *Classification and Diagnostic Criteria of Mental Disorders in China* formally deleted homosexuality from the psychosis category. These developments promoted a “social environment liberalisation and identity formation” among Chinese homosexuals (Huang, 2018, p. 344). However, two decades later, it is still common to regard people with same-sex orientation (henceforth PWSO) as abnormal. An alarming example was a recent publication by Jinan University Press (henceforth JUP). In 2016, the JUP published a textbook titled *Mental Health Education for Chinese University Students*. It classified homosexuality as one of four psychosexual disorders, together with transvestism, fetishism, and voyeurism. This classification caught the attention of Xixi, a student who identified as a lesbian. Xixi took legal action against JUP on 6 July 2017, after negotiations failed, demanding a public apology and an end to the stigmatisation of homosexuality. A court in the Chinese city of Suqian accepted Xixi’s lawsuit on 17 July. Hearings on the case began on 28 July 2020, but the judgment pronounced on the 2nd of September was unfavourable to Xixi. On 24 February 2021, the court’s earlier ruling was upheld after an appeal.

The failure of Xixi’s lawsuit against JUP’s homophobic textbook sparked fierce reactions on China’s Sina Weibo and even topped the 3 September 2020 Weibo *reso*^1^. Such a phenomenon is unusual on Chinese social media, as it is challenging to get a controversial topic like homosexuality onto the Weibo *reso*. The verdict was dividedly supported by some who saw it as a defence of the nation, and opposed by others who saw it as a threat to civil liberties.

Since little research has been done on representations of PWSO on Chinese social media, this study aims to fill the gap by analysing the Weibo comments^2^ supporting the verdict. We are specifically interested in the discursive representation of homosexuality and the major sociocultural factors that influence the patterns of this representation. It extends the theoretical application of van Leeuwen’s (2008) delegitimation theory to study gender and sexuality issues in a non-Western context.

## The representation of homosexuality in China

The belief that PWSO in contemporary China have not yet reached full rights equality is widely recognised (Wu, 2003). As in most Asian countries, it remains challenging for Chinese PWSO to gain wider social acceptance. Compared to more LGBTQ-friendly countries, Chinese queer culture is only slowly gaining more visibility in Pride events (Markwell and Waitt, 2009; Milani, 2015), public LGBT posters and art performances, and gay bars or bathhouses. Thanks to the concerted efforts of medical experts and gay activists (Kang, 2012), since 2001, homosexuality is no longer officially considered a mental illness in China. In addition, an increasing number of scholars, gay rights advocates, and self-declared PWSO have demanded that the Chinese Party-State consider legalising same-sex marriage (Liu and Zhu, 2020).

Despite this progress, the government’s political agenda does not include the rights of PWSO (Zhang, 2014). Even though discriminatory discourse about LGBTQ individuals became more covert (Nartey, 2022), it is still easy to find instances of Chinese PWSO being negatively portrayed (see Kang, 2010; Huang, 2018).

Based on two urban tabloids from 1900 to 1949,^3^ Kang (2010), for instance, explores two themes: emperor-male favourite relationships and the (de)legitimisation of same-sex acts. The first theme is a retelling of the Lord Longyang love affair following the fall of the Qing Dynasty, focusing on two male couples: Beijing warlord Cao Kun and Li Yanqing; and Puyi, the last Qing emperor, and his eunuchs. Such sexually deviant relationships, according to the author, would have caused a political crisis and weakened the nation. Kang notes, however, that Qing Dynasty criminalised same-sex relationships more stringently than its successor, the People’s Republic of China. According to our knowledge, Kang’s (2010) study was the first to examine Chinese media coverage of homosexuality. Zhang (2014) extends Kang’s (2010) research by conducting an analysis of news coverage of homoeroticism spanning from 1949^4^ to 2013, a period witnessing the death and rebirth of Chinese homosexuality. Zhang makes several interesting observations. Firstly, during the Maoist period, homoeroticism was politically and culturally repressed and seen as a vice of the capitalist West. This perception changed somewhat after 1978. Secondly, homoeroticism was presented as the sole source of AIDS, a “capitalist disease.” And thirdly, homoeroticism, which stemmed from Western capitalist individualism, was supposed to clash with China’s socialist collectivism.

Chang and Ren (2017) carry out a critical discourse analysis of representations of Chinese homosexuals in five urban tabloids. Four portrayals can be distinguished. First, homosexuals are portrayed not just as victims of crime (gay as victims of robbery and blackmail, lesbians as victims of rape) but also as criminals who threaten China’s social stability. Also, they threaten to destroy traditional marriage. The reason why lesbians are more accepted than gay men in China is that they are seen to be more akin to heterosexuals and hence less perilous to society.

Recent research by Wang and Ma (2021) uses Chinese English-language newspapers to investigate media portrayals of homosexuality. However, the focus of their research is not so much the gay community as the issues concerning its members, such as Chinese sex education programmes, LGBTQ+ civil rights, and the removal of homosexuality-related content from Chinese media. Slightly different from previous research, the coverage of such issues in recent years shows indications of more progressive and liberal attitudes toward LGBTQ+. Despite this, news articles continue to associate HIV/AIDS with homosexuality.

These studies have depicted PWSO in Chinese newspapers (1900–2018) using a linguistic, sociological, or discourse approach. Nonetheless, two significant gaps still remain. As a consequence of their exclusive focus on print media discourse, they disregard other vehicles, like television and social media. And second, individual and non-institutional attitudes toward Chinese PWSO have not received much attention, as most existing research focuses on institutional and official points of view. With these lacunae in mind, we explore how ordinary Chinese (i.e., social media users) use language to delegitimise homosexuality. Our study was guided by the following research questions (RQs):

RQ1: How is homosexuality discursively represented on Sina Weibo?

RQ2: What sociocultural factors underlie the patterns of representation identified?

### Theoretical framework

To examine the discursive delegitimisation of homosexuality in comments on Weibo, we draw basically on van Leeuwen (2008). According to his analytical framework, legitimation serves to provide “good reasons, grounds or acceptable motivations for past or present action” (Van Dijk, 1998, p. 255). It consists of four broad categories of legitimation of social practices: authorisation (via the authority of people, traditions, customs, and countries), moral evaluation (through the value systems of society as expressed through evaluation, abstraction, and comparison), rationalisation (through truth and reality), and mythopoesis (through narratives or storytelling). Each legitimation category can be further subdivided into subcategories and used separately or in combination with others.

This legitimation theory can be extended to delegitimise particular social acts. According to van Leeuwen (2008), each category “can be used to legitimise, but also to delegitimise” practices and institutions (p. 106). By delegitimising, social actors or actions are consistently portrayed as controversial, unethical, troublesome, deviant, or undesirable (see Ross and Rivers, 2017; Tiainen, 2017; Ross, 2020). Yu’s (2022) article on Chinese single women and Liu’s (2021) research on the legalisation of same-sex marriages in Taiwan broaden van Leeuwen’s (2008) theory of gender and sexuality. Their research focuses on the characteristics of negative-other representations and supports the applicability of the delegitimation theory in critical discourse analysis, providing a foundation for the present study.

## Materials and methods

We collected the data for this study on Sina Weibo by using a specific hashtag, #The Case of Homophobic Textbook (*kongtong jiaocaian* 恐同教材案)#. Starting in 2009, Sina Weibo is a Chinese social media platform similar to Facebook and Twitter, where users can “post their life, discuss their idols and comment on social issues” (Jin and Chen, 2020, p. 3) and also repost others’ messages. We chose Weibo as our data source for two reasons. First, Weibo acts as a dialogical network (Leudar and Nekvapil, 2004) in which Chinese netizens can comment on hotspot issues such as the Xixi case anytime, anywhere. Second, we can think of Weibo as a “digital culture” that bridges the “online and offline worlds” (Wiggins, 2019, p. 23). Despite censorship, users on Weibo are free to express themselves and reply to one another. Users support or oppose contentious issues and believe that their opinions matter. Weibo users form different social groups by reposting or liking comments. Regarding the ethical aspects of this study, following Ho (2020), we see Weibo comments as publicly and anonymously posted “opinions and information,” so “there are no ethical concerns” in our case study (p. 51).

While a Weibo news item can generate considerable discussion, other news stories frequently replace it the following day. Considering this, we targeted an 18-h period beginning at 21:17 on 3 September 2020, when the first comment was posted on Sina Weibo, to collect the comments generated by the Xixi case. The collection process consisted of two steps:

Step 1: The lead researcher downloaded all 1,009 comments posted within the aforementioned time filter.
Step 2: Two additional researchers collaborated to cluster the collected comments and eliminate 513 for the following reasons. First, Weibo comments (*n* = 31) unrelated to the Xixi case or homosexuality were eliminated. Second, neutral (*n* = 67) and pro-homosexuality comments (*n* = 415) were discarded because this research investigates the nature of public opposition to homosexuality.

In the end, we were left with 496 homophobic comments. As Coffey and Atkinson (1996) suggested, we categorised these comments using a thematic analysis approach. Firstly, each comment was independently coded based on its “general thematic content” (Coffey and Atkinson, 1996, p. 35). Before analysing all comments in detail, we clustered related codes into thematic categories such as causes (reasons for same-sex desires), effects (consequences of homosexuality), attributes (characteristics attributed to homosexuality), and responses (measures to address homosexuality). Subsequently, we reread the classifications together. When divergent opinions arose, a third party was consulted to make the final decision. Below is an illustration of our analytical procedure.

**Weibo 1**

大陆是绝对不可能的, 在中国大陆, 国家都觉得是一种病, 民众还能咋办呢

“Homosexuality is not possible in mainland China, where even the government thinks it’s a disease. What can the public do?”

In Weibo 1, the lexical choice of “government” (*guojia* 国家) shows an impersonal authority. However, other strategies are used here, namely (1) comparing homosexuality to disease and (2) explaining the government’s influence on public opposition to homosexuality. Therefore, we coded this comment as a mixed strategy involving impersonal authorisation, comparison, and explanation. The author of this comment appears to delegitimise homosexuality on the basis that the government has entitled citizens to do so. During this process, we also noticed that some Weibo comments were more popular and typical than others, such as Weibo 13, which received 38 likes. This observation reminded us to select representative samples to better illustrate discursive representations of social actors and practices (Liu, 2021).

## Results

Table 1 shows the number of realisations of delegitimisation strategies on Sina Weibo. The most frequently used strategy is authorisation, accounting for 37.8%. This is mainly because Weibo users often emphasise their views on traditional values and beliefs to make their comments sound more reasonable and convincing (e.g., Weibo 13). They also delegitimise homosexuality by appealing to an impersonal authority, as in “It’s impossible for Xixi to win the lawsuit because the government has not yet recognised homosexuality.” The second most common strategy is rationalisation (32.6%), most of which comes from theoretical rationalisation. It is typical for users to explain why homosexuality should not be legalised in China. Some users prefer to present the consequences of legalising homosexuality, “the legalisation of homosexuality will only lead to the extinction of the human species.” Rationalisation is followed by moral evaluation in 28.1% of cases. Weibo comments are characterized by comparisons that are used to negate homosexuality, such as referencing nations that have recognized same-sex marriages. Users also use evaluative nouns and adjectives, as in “homosexuality is anti-human” and “why do we say homosexuality is normal.” The least used strategy is mythopoesis (1.4%). Weibo users employ this strategy to describe the adverse effects of homosexuality.

**TABLE 1:** Frequency of each delegitimation strategy.

Delegitimation strategy	Subcategory	Total	Frequency (%)
Authorisation	Personal	164	8.8%
	Impersonal	225	12.1%
	Tradition	217	11.7%
	Conformity	23	1.2%
	Expert	34	1.8%
	Role model	41	2.2%
Moral evaluation	Evaluation	245	13.2%
	Abstraction	127	6.8%
	Analogies	151	8.1%
Instrumental rationalisation	Goal orientation	54	2.9%
	Means orientation	12	0.7%
	Effect orientation	142	7.6%
Theoretical rationalisation	Definition	164	8.8%
	Explanation	173	9.3%
	Predictions	47	2.5%
	Experiential rationalisation	0	0
	Scientific rationalisation	14	0.8%
Mythopoesis	Moral tales	0	0
	Cautionary tales	26	1.4%

This section attempts to respond to the first research question by situating van Leeuwen’s (2008) discourse model within the Chinese context. However, for lack of space, we will present only four themes identified on Weibo to illustrate the discursive delegitimisation of homosexuality. Specifically, homosexuality has been represented as unhealthy (i.e., it is one type of mental and physical diseases to cure), infertile (i.e., it threatens global population growth and violates China’s pronatalist agenda), disruptive (i.e., it endangers the continued stability and prosperity of Chinese society), and corrosive (i.e., it runs counter to and challenges established traditions and norms in China).

### Homosexuality as a mental and physical illness

“Disease” is the most prevalent argument for delegitimising homosexuality. This is a typical newspaper practice (Zhang, 2014; Chang and Ren, 2017). The disease discourse constructs PWSO as a mental or physical illness. In particular, moral evaluation is found to have a higher frequency when delegitimising homosexuality as a social practice, whose abstraction strategy is repeatedly used when distilling moral qualities such as threat (Weibo 2), abnormality (Weibo 3), and illness (Weibo 6).

**Weibo 2**

同性恋应该属于一种怪癖吧, 就和恋童癖, 恋妇癖, 恋物癖都差不多

“*Tongxinglian*^5^ resembling paedophilia, Oedipus complex, and Fetishsm should belong to the same category of eccentricity.”

**Weibo 3**

正常, 拼音 *zhèng cháng*, 指一个人在做事中, 要顺应客观规律的发展, 与实际常人应该做的一致。我是同等对待, 没有歧视, 但同性恋显然属于 “非正常” 的。

“Normal, pronounced *zhèng cháng* in Chinese, means that when embarking on a project, one should abide by the development of objective laws and follow what normal persons normally do. While I treat homosexuality and heterosexuality as equal, and do not discriminate against homosexuals, the former is obviously abnormal.”

In Weibo 2, homosexuality is negatively associated with other sexual perversions. Notable is the use of the modal verb “should” (*yinggai* 应该), which conveys the moralistic belief that homosexuality is harmful to normal people. As demonstrated on Weibo 3, homosexuality is regarded as deviant behaviour. Its author asserts that PWSO’s actions have veered off course, provoking public criticism and opposition. This finding is consistent with Blommaert and Verschueren (1998, p. 35): “If you aren’t within the normal range, you are in a sense abnormal, and may be pitied, medicalised, ostracised or criminalised.” In a few societies, such as Thailand (Fongkaew et al., 2019) and the UK (Baker, 2005), homosexuality has been medicalised as a venereal disease, particularly in connection with HIV transmission. Weibo 4 defines homosexuality as a mental illness based on the results of a “scientific” experiment. By attributing his speech to scientific research, the author increases his illocutionary force and avoids responsibility.

**Weibo 4**

之前MIT和哈佛联合做了一项研究, 基于47万人 (同性恋) 的遗传信息进行了全基因组关联分析 (GWAS), 找不到同性恋的遗传学解释。对不起, 就目前的研究结果来说, 同性恋就是心理疾病。

“Some time ago, MIT and Harvard University carried out collaborative research. However, they failed to find explanations for *tongxinglian* from a genetic perspective, based on a genome-wide association study of the genetic information of 470,000 *tongxinglian*. I regret to say that, as far as the current research is concerned, *tongxinglian* is definitely a mental illness.”

**Weibo 5**

你喜欢谁是你的自由, 但是作为一个前疾控中心工作人员, 我说实话, 如果男同少一些, 那么每年本市的hiv阻断药比如特威凯就可以节省七成以上。

“It is your freedom to love anyone. However, as a former staff of the Centre for Disease Prevention and Control, I have to tell the truth that if there were fewer gay men, the use of HIV blockers, Dolutegravir Sodium included, would decrease by over 70% each year.”

In addition to being viewed as a psychological deficiency, homosexuality is viewed as a physical illness, particularly in connection with AIDS. Delegitimising homosexuality, Weibo comments are primarily made through individuals (given their professions, educational backgrounds, roles, and social status). Weibo 5 is a striking example of the use of personal authority, as its author reinforces his or her opposition to homosexuality by explicitly appealing to his or her former hospital staff experience. By revealing the “truth,” this author implies that the fewer PWSO there are, the more HIV “treatment” medication could be saved. The implication here recalls Reddy’s (2002) finding that it has been reported that homosexual men transmit not only their sexual orientation but also disease.

Analogous to Weibo 5, the public in China is constantly reminded that PWSO are HIV carriers (see Weibo 6). Chang and Ren (2017) state that homosexuality has been grouped with other social ills, such as AIDS and drug abuse, being a threat to Chinese social stability. Huang (2018) notes that only recently homosexuals are no longer considered the sole source of HIV/AIDS transmission. In response to this stigmatisation, Weibo 6 employs what van Leeuwen (2008, p. 37) calls “aggregation,” by which PWSO who transmit AIDS are quantified to elicit an emotional response from the public.

**Weibo 6**

赶快去查一下国内传播艾滋等病占比最高的传播方式是什么

“You’d better hurry up and find out which mode of transmitting diseases, such as AIDS, is the highest in mainland China.”

### Homosexuality as a threat to population growth

Homosexuality is also portrayed as a disruptive influence on the global population and Chinese society. Homosexuality is frequently mentioned in the context of “human beings” (*renlei* 人类), “survival” (*shengcun* 生存), “human reproduction” (*fanyan* 繁衍; *fanzhi* 繁殖), and “offspring” (*houdai* 后代). At the same time, PWSO on Weibo are described as “destroying the world,” “unbalancing the harmony between men and women,” and “failing to give birth to children.” The following two Weibo comments serve to illustrate this.

**Weibo 7**

但繁衍又是最重要的, 为何日本, 欧洲对其低生育率忧心忡忡。按你这么说, 他们都是无病呻吟呗

“However, human reproduction is still the most important issue. Why do Japan and Europe worry about their low fertility rates? In your view, they are only making a fuss about an imaginary illness.”

**Weibo 8**

同性恋如果影响到人类繁衍 那就是病毒 如果没有 可以包容

“If *tongxinglian* has negative impacts on human reproduction, then, of course, it is a virus. If not, we can tolerate it.”

Weibo 7 explains why homosexuality should be delegitimised. Starting with the adversative word “however” (*dan* 但), this comment first refutes the other’s position by mentioning human reproduction and then citing other countries to support its claim. It also uses a cultural idiom, “make a fuss about an imaginary illness,” emphasising that increasing the birth rate should be a priority to maintain social stability. This gives the impression that children are more important than individual desires. Weibo 8 appears to send a positive message about the proper way to view homosexuality. For this author, homosexuality is acceptable as long as it does not harm people’s wellbeing. However, the author does not evade the opposition to homosexuality by explaining his or her position.

Significantly, what these commentaries have in common is that they delegitimise homosexuality by creating a “homosexual panic” (Reddy, 2002, p. 171) and misleading other commenters. It is common knowledge that China’s population is ageing. To counter this trend and increase the birth rate, the Chinese government has adopted a series of policies, including the two-child policy in 2015 and a three-child policy in 2021. Despite these measures, in 2020, the fertility rate was at its lowest since 1978.^6^ This context may contribute to making homosexuality the scapegoat for the lack of children.

**Weibo 9**

虽然不歧视同性恋但绝对鲜明的表示反对同性恋, , 不知道是那个国家开始大张旗鼓地公开支持这种行为, 但从自然界的进化来说, 这肯定是一种病态, 如果人类和其他动物界都是这种状态, 人类如何生存传承??

“While I do not discriminate against *tongxinglian*, I strongly oppose them. It is not clear which country was the first in the world to support the same-sex act openly. However, homosexuality must be a disease based on the evolution of nature. If human beings and the animal kingdom were trapped in this act, how could we humans survive??”

In order to stir up the panics surrounding homosexuality, Weibo users prefer combining mythopoesis with other delegitimisation strategies. Weibo 9 is an example. The structure of this comment can be broken down as follows: (1) Attitude. This commenter opposes homosexuality but argues that his/her opposition does not imply that he/she supports discrimination against PWSO; (2) Definition. Based on the theory of evolution, homosexuality can be considered a disease. Therefore, same-sex love is a kind of behaviour and not an identity. Likewise, Baker (2005) found that homosexuality in British parliamentary debates is frequently associated with “external acts or behaviours” as opposed to an identity (p. 44). Similarly, Liu (2021) notes that the younger generation uses concerns about the copying of homosexual behaviour to attack the legalisation of same-sex marriage in Taiwan; and (3) Illusion. Heterosexuality, in turn, is portrayed as being responsible for the wellbeing of human beings and the survival of the world. PWSO may corrode and endanger the world and future generations of Weibo users, endangering the survival of humanity. This kind of delegitimisation by mythopoesis is evident in claims that the future of humans and families and the fate of any society cannot depend on same-sex love but only on heterosexual marriages (Kania, 2020). This again legitimises the portrayal of homosexuality as responsible for population decrease.

### Homosexuality as a danger to Chinese society

A third prominent theme is homosexuality as a (potential) risk for China as a society. Weibo comments frequently use analogies comparing same-sex attraction to other behaviours associated with negative values. That is, homosexuality is always compared to “red-light districts.” (*hongdengqu* 红灯区), “gambling” (*dubo* 赌博), “murder” (*sharen* 杀人), and “marijuana” (*dama* 大麻). “Sects” (*xiejiao* 邪教) in Weibo 10 are rejected by most governments worldwide because of their mind-controlling, family-destroying and socially destabilising characteristics. Similarly, homosexuality is excluded from existing laws in mainland China because of its deviant and unacceptable characteristics. As China has tried to build a harmonious society, homosexuality is not tolerated. Comments associate homosexuality with illegal activities and sexual perversion (see Weibo 2), giving the impression that homosexuality is dangerous.

**Weibo 10**

是的, 跟邪教似的, 还得让法律承认他们, 奇怪。

“Yes, homosexuals are like members of a heretical sect. It is absolutely strange to demand that Chinese laws recognise them.”

What also stands out in Weibo 10 is the keyword “laws” (*falu* 法律), as this word has a high frequency on Weibo. Chinese laws are not intended to serve a specific individual or group, such as the LGBTQ+ community, but to promote social stability and prosperity. The more aggressive PWSO are, the more panic among the public. In Weibo 11, for example, the commenter expresses concern about the possible official recognition of homosexuality by using the subjunctive, indicating that legislative changes will affect the current legal system. The same strategy is used in Weibo 12, which claims that legalising homosexuality is only a first step and will lead to a demand to have children through surrogacy. Given that “homosexual rights have not entered the [political] agenda of the Chinese government” (Zhang, 2014, p. 1019), such statements appear to be based on the assumption that China’s existing laws are in a state of disarray. However, these Weibo comments contribute to the recent observation that the impersonal authority of laws is frequently used to delegitimise same-sex relationships (Oyebanji, 2023).

**Weibo 11**

这特么要是同性恋合法了, 得顺带建立多少奇奇怪怪的法律来保障他们这群人的权利, 新颁布的民法典估计要从头改到尾

“If fucking *tongxinglian* were to be legalised, there would be more strange laws to protect the rights of these people. If so, the recently enacted Civil Code of the People’s Republic of China is likely to be thoroughly amended.”

van Leeuwen (2008) divides delegitimation by mythopoesis into moral and cautionary narratives. The former praise legitimate social practices, while the latter warn that non-normative actions will be punished. Several scholars (e.g., Cheng, 2021; Yu, 2022) find that, compared to other strategies, this type of strategy is used less in institutional or official discourse. However, Liu (2021) demonstrates that narratives emphasising negative consequences dominate the grassroots discourse. His conclusion holds for our Weibo samples, as evidenced by Weibos 11 and 12.

**Weibo 12**

虽然政治不正确, 但我国男同可千万别合法, 男同合法下一步就该嚷嚷要代孕合法了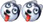.

“While it is politically wrong to proscribe homosexuality, Chinese homosexuality should not be legalised. If legalised, gay men will then call for surrogacy to be legalised in China.”

### Homosexuality as a rebellion against traditional cultures and values

Additionally, Weibo commenters delegitimise homosexuality by characterising it as contradicting Chinese tradition, ranging from cultural beliefs to core socialist values. Regarding the Xixi case, Weibo users who delegitimise homosexuality lean heavily on the authorisation strategy. Their comments refer to an impersonal authority focusing on “traditions” (*chuantong* 传统), “public opinion” (*mini* 民意), “culture” (*wenhua* 文化), “national and cultural characteristics” (*growing* 国情), “laws” (*falu* 法律), “Chinese civilisation” (*huaxia wenming* 华夏文明), and “Chinese characteristics” (*zhongguo tese* 中国特色).

**Weibo 13**

中国的文化一直都是阴阳结合, 这才是正常的, 西方文化已经走偏, 少数人的利益绑架了大多数人, 同性恋在任何社会都是极少数人, 但是政治正确却让这部分人可以影响整个社会

“Chinese culture has always been a combination of darkness and lightness (i.e., feminine and masculine), which is normal and acceptable. Western culture has failed because the interests of the minority have kidnapped those of the majority. *Tongxinglian* are a minority in every society. However, the political correctness (i.e., legalising *tongxinglian*) will ensure that these people will influence the whole society.”

**Weibo 14**

我们国家把生殖看的很重要, 在老一辈的观念里传宗接代是一件大事, 所以我觉得同性恋在我国终究不会合法。

“Our country attaches great importance to producing offspring. For the older generations in China, continuing the family bloodline with legitimate offspring is of utmost importance. That is why, for me, *tongxinglian* in mainland China will never be legalised.”

Weibo 13 is a notable example in which “culture” functions as a keyword. A family metaphor appears here, where “darkness” (*yin* 阴) corresponds to the woman (as a wife), while “light” (*yang* 阳) refers to the man (as a husband). Traditionally, a Chinese family consists of a woman and a man, which implies heterosexual marriage is considered as the norm in China. Liang et al. (2016) corroborate this by noting that Chinese textbooks have repeatedly advocated that in a healthy “family landscape” (p. 114), children should be supported by female-male parents rather than same-sex parents. Examining the public discourse around homosexuality, similarly, Ivanova (2018) concludes that homosexual relationships in Tanzania are seen as “meaningless” because they cannot bear children. Her observation is in line with the Chinese context, where Weibo users invoke traditional Chinese customs, such as “ to get married and own a business” (*chengjia liye* 成家立业) and “ to raise children to continue the family line” (*chuanzong jiedai* 传宗接代), to delegitimise homosexuality. As illustrated in Weibo 14, the need for procreation stands in the way of legalising homosexuality. The keyword “offspring” is repeated to emphasise that nothing is more important in China than having children.

As for Weibo 15, the lexical choice of “patients” (*huanzhe* 患者) and “normal people” (*zhengchangren* 正常人) are noteworthy, given that the commenter explicitly legitimises his/her perception of homosexuality as a disease. Moreover, he/she repeatedly uses rhetorical questions to emphasise the support for preserving Chinese characteristics and uniqueness, indicating the opposition to legalising same-sex marriages. Similarly, Weibo 16 questions readers by asking them to consider whether they will accept substances that are considered legal abroad but illegal in China. Through comparisons and evaluations, homosexuality is represented as pathetic, as it is not in line with Chinese traditions.

**Weibo 15**

我觉得第一个, 其他国家把同性婚姻合法化中国就要跟着走吗? 其他国家废除死刑中国也要跟着废吗? 西方价值观下就一定是正确的吗? 第二个, 强迫其他人把同性恋患者当正常人看待才是不平等

“First, should China follow the example of other countries to legalise same-sex marriages? Will China follow other countries in abolishing the death penalty? Are Western values really right? Second, forcing others to regard *tongxinglian* patients as normal persons is unfair.”

**Weibo 16**

国外的就一定正确? 荷兰允许红灯区, 美国不许堕胎, 澳门允许赌博, 你同意在你周边做这些事吗?

“Are social practices abroad really good? Netherlands have allowed red-light districts. America does not allow abortion. Macao allows gambling. Do you agree that these things happen on your side?”

## Discussion

In relation to RQ1, the preceding analysis indicates that the general public employs Weibo as a participatory platform to stigmatise and delegitimise PWSO by conveying negative sentiments toward homosexuality. As for RQ2, within negative comments regarding homosexuality and PWSO, the following three sociocultural topics stand out:

### Nationalism on Weibo

Nationalism is one characteristic of Weibo comments on PWSO. It is typical of nationalist sentiment “to occupy online spheres (where possible in alliance with netizens) with discourse couched in nationalistic language” (Hänska et al., 2020, p. 578). Homophobic Weibo users evaluate themselves positively and others negatively, as shown in the analysis (Weibos 15 and 16). They are also cautious about marginalised groups using “political correctness” for their own gain. Weibo users label same-sex relationships as negative social issues, enhancing moral panic (Litosseliti, 2007). These users view homosexuality as a Western phenomenon. As Liu (2021) shows, homophobic ideologies and nationalist values often overlap in China, reinforcing each other. In recent years, similar phenomena have been observed elsewhere. For example, homophobic individuals in Poland view LGBT communities as threats to Polish national values (Żuk and Żuk, 2020).

### Collectivism on Weibo

For centuries, China has prioritized collectivism. China’s collectivism is like the Great Wall; individualism is the bricks used to build it. A person who pursues himself and exhibits personality is incompatible with Chinese mainstream values and may get perceived as the challenge of and threat to the collectivism. For example, woman who has reached a marriageable age but fails to get married due to self-pursue of freedom has been ironically called leftover woman or a social problem (Yu, 2022). Similar to the leftovers, PWSO are criticised by netizens in a climate of pervasive collectivism. In the case of Xixi (e.g., Weibos 11 and 12), users associate the rise of homosexuality with a dark future and legalising same-sex marriages and securing other rights for PWSO will erode China’s social collectivism. Therefore, they suppress individual desires by arguing that the self-improvement must contribute to China’s development. For them, this helps to align individuals with the Chinese nation.

### Patriarchy on Weibo

Patriarchy predominates in netizens’ criticisms of PWSO. On Weibo, patriarchy is mainly expressed by getting married and producing offspring. The value of marriage has a high frequency on Weibo. Heterosexual marriage in China connects not only two people but also two families and their social networks. Failure to marry at the appropriate age is abnormal and deviant for failing to extend the family network (Chou, 2001, p. 34) and brings dishonour to the whole family. Under pressure, PWSO choose to marry heterosexual or lesbian women. Close family ties increase the chances of PWSO marrying heterosexual women (Shi et al., 2020). In other words, PWSO with greater filial piety are more likely to enter heterosexual relationships (Wen and Zheng, 2020).

An additional analysis of patriarchal Weibo remarks centres around a particular slogan, specifically “legitimate offspring” as it pertains to the “family lineage” (Wu, 2003, p. 118). This is reflected in traditional beliefs such as *buxiao yousan*, *wuhou weida* (there are three unfilial acts, the worst of which is to have no offspring) and *chuanzong jiedai* (raising children to continue the family line). Compared to heterosexuals, however, it is more difficult for PWSO to fulfil such filial duties, as the promotion of same-sex marriage and adoption by PWSO in mainland China still “remains more slogan than reality” (Wang and Ma, 2021, p. 198). To prevent the family from losing face, PWSO have three options, the first of which concerns marriage as mentioned above. The second option is to pay for surrogacy, while the third is to adopt children. For Weibo users, the interplay of getting married and producing offspring means they should fulfil their family duties. Chinese men, for example, are expected to work outside the home and care for their wives and children at home. However, as evidenced by Weibos 13 and 14, PWSO are accused of violating this principle and undermining Chinese traditions.

## Conclusion and limitations

By analysing the way in which the general public delegitimises homosexuality on the Weibo platform with the Xixi case as a starting point, this article contributes to the extant scholarship. Using van Leeuwen’s (2008) delegitimation strategies, such as authorisation, moral evaluation, rationalisation, and mythopoesis, this article demonstrates how Weibo users oppose the legalisation of homosexuality. In particular, users tend to portray homosexuality as (1) a mental and physical illness, (2) a threat to population growth, (3) a danger to Chinese society, and (4) a rebellion against traditional values. This article sheds light on notable sociocultural factors (i.e., nationalism, collectivism, and patriarchy) that foster anti-homosexual sentiments among Weibo users, resulting in negative representations.

While the delegitimation framework helps to explain how the population delegitimises homosexuality, readers may benefit from more discussions on how values rooted in the Chinese socio-political environment impact the delegitimisation of homosexuality. Moreover, given that punctuation marks, images, and emojis as examples of mode can generate meanings in representation and communication (Kress, 2009), “any form of text analysis which ignore [these] will not be able to account for all the meanings expressed in texts” (Kress and Van Leeuwen, 1998, p. 186). In this sense, it would be worth analysing the semiotic devices used on Weibo, such as the emojis in Weibo 12. Finally, this article presents only a tiny part of the Weibo comments generated by the Xixi case. The investigation has been limited to Weibo comments that oppose homosexuality, while comments that support homosexuality have been ignored. Because some Weibo commenters self-identify as lesbian or gay, it would be interesting to listen to their voices to comprehend why they advocate for legalising homosexuality in China.

Despite these limitations, this study illustrates how ordinary people view homosexuality in China. At the same time, this study extends previous research on delegitimation in political discourse (Ross and Rivers, 2017; Tiainen, 2017) to sexist discourse, echoing Ross and Rivers (2019) argument that new media “aid in conveying a particular belief or ideology or in engaging with the participatory culture” (p. 9).

## Data availability statement

The original contributions presented in this study are included in the article/supplementary material, further inquiries can be directed to the corresponding author.

## Author contributions

KZ: conceived the original idea, carried out the research, and drafted the manuscript. HZ: helped to supervise the research. Both authors contributed to this article, revised the manuscript, and approved the submitted version.

## Footnotes

1 This refers to the ranking of daily trending topics.

2 Using language to resist and delegitimise homosexuality in China.

3 This is a transitional era from a dynastic China to the Republican government of the Kuomintang.

4 The People’s Republic of China was founded in 1949.

5 It is an indigenous term referring to the same-sex love.

6 Available from https://zhuanlan.zhihu.com/p/436013343.
